# The Relationship Between Collateral Circulation and Electrocardiographic Frontal QRS-T Angle in Patients with Coronary Artery Chronic Total Occlusion

**DOI:** 10.3390/jcm15010148

**Published:** 2025-12-24

**Authors:** Muhammet Öztürk, Nadir Emlek, Ali Gökhan Özyıldız, Elif Ergül, Hüseyin Durak, Afag Özyıldız, Mustafa Çetin

**Affiliations:** 1 Department of Cardiology, Faculty of Medicine, Recep Tayyip Erdoğan University, Rize 53100, Turkey; muhammet.ozturk@erdogan.edu.tr (M.Ö.); nadir.emlek@erdogan.edu.tr (N.E.); elif.ergul@erdogan.edu.tr (E.E.); huseyin.durak@erdogan.edu.tr (H.D.); mustafa.cetin@erdogan.edu.tr (M.Ç.); 2 Department of Cardiology, Training and Research Hospital, Recep Tayyip Erdoğan University, Rize 53100, Turkey; drafagj@gmail.com

**Keywords:** frontal QRS-T angle, collateral circulation, chronic total occlusion

## Abstract

**Background**: Ischemic heart disease is the primary contributor to global mortality. The QRS-T angle at the anterior aspect of the heart serves as a significant biomarker of the heterogeneity in myocardial repolarization and the electrophysiological instability of the cardiac myocytes. A wide frontal QRS-T angle is associated with proximal vascular disease, coexistence of three-vessel disease, and increased mortality. Hereby, we aimed to examine the relationship between collateral circulation and frontal QRS-T angle in patients with chronic total occlusion (CTO). **Methods**: A cohort comprising 120 patients (17 females, 14.1%) who received a diagnosis of chronic total occlusion (CTO) subsequent to the administration of coronary angiography conducted for the evaluation of stable angina pectoris was incorporated into the investigation. The electrocardiographs of the patients were evaluated in detail, and the frontal QRS-T angle was calculated. The patients were categorized into two groups: subjects exhibiting an increased frontal QRS-T angle (>110° for men, >90° for women) and those presenting with a normative frontal QRS-T angle. Coronary angiographies of the patients were analyzed, and coronary collateral circulation was classified according to Rentrop classification. **Results**: Serum albumin level (OR = 0.711, 95% CI 0.564–0.896; *p* = 0.004) and poor collateral flow (OR = 17.7, 95% CI 12.2–85.3; *p* < 0.001) were significant predictors of raised frontal QRS-T angle. **Conclusions**: The frontal QRS-T angle is a novel parameter that is more reliable, consistent, and less sensitive to miscalculation and misidentification than other conventional electrocardiographic myocardial repolarization parameters. Revealing the bad collateral relationship with the frontal QRS-T angle may enable physicians to take more stringent precautions and change the risk factors related to the increased QRS-T angle in advance.

## 1. Introduction

Coronary artery disease (CAD) is a prominent contributor to both death and illness on a global scale. In the context of coronary angiography (CAG), which is a diagnostic procedure utilized to visualize the blood vessels of the heart, it has been observed that the incidence rate of chronic total occlusion (CTO) approaches a noteworthy percentage of 15%. This rate rises to 20% in patients who have been formally diagnosed with CAD, suggesting a strong correlation between the presence of CAD and the likelihood of encountering CTO during angiographic evaluations [1]. Well-developed collateral circulation (CC) serves as a safeguard in CTO. Collateral circulation is vascular structures formed as a chronic response within the coronary artery itself or between different coronary arteries in order to maintain the blood supply and vitality of the myocardial tissue distal to the lesion in case of critical stenosis or total occlusion that disrupts the circulation [2]. A well-developed coronary collateral circulation (CC) plays a crucial role in the prevention of ischemic events, which is a condition characterized by insufficient blood supply to the heart, and, furthermore, it has been demonstrated to be significantly effective in diminishing the size of the infarcted area, consequently thwarting the formation of left ventricular aneurysms, enhancing the functional capacity of the left ventricle following an infarction, decreasing the incidence of mortality associated with cardiovascular diseases, and ultimately contributing to an increase in long-term survival rates among affected patients [3,4,5,6].

The frontal QRS-T angle is regarded as a novel indicator of myocardial repolarization, characterized by the disparity in angle between the direction of ventricular depolarization (QRS wave) and ventricular repolarization (T wave) [7]. The frontal QRS-T angle can be readily calculated in 12-lead electrocardiography (ECG). The frontal QRS-T angle increases in conditions such as hypertension, left ventricular concentric hypertrophy, heart failure, coronary artery disease, anemia, coronary slow flow, and left ventricular diastolic dysfunction [8,9,10,11,12,13]. Mortality is higher in patients with cardiovascular disease with an increased frontal QRS-T angle than in individuals with a normal frontal QRS-T angle [14]. This study aimed to examine the correlation between the existence of poor collaterals and frontal QRS-T angle in patients diagnosed with CTO.

## 2. Materials and Methods

The present investigation is a single-center, observational, cross-sectional study. A comprehensive cohort consisting of 163 individuals who underwent the intricate medical procedure of diagnostic coronary angiography during the specified time frame spanning from April 2023 to December 2023, primarily due to the manifestation of stable angina persisting for a minimum duration of three months, was meticulously selected for inclusion in this study; these individuals were subsequently diagnosed with total occlusion in at least one coronary artery and were clinically assessed to possess chronic total occlusion (CTO) based on both clinical evaluations and angiographic findings. Within this cohort of individuals, a selection of 43 patients were systematically excluded from the study based on the exclusion criteria. Consequently, the remaining population of 120 patients was identified as having stable angina directly attributable to a chronic total occlusion lesion, and these individuals subsequently advanced to participate fully in the study as designed.

The patient’s anamnesis, physical examination, 12-lead ECG, echocardiography and CAG findings, systemic diseases, and medications were recorded. The calculation of body mass index (BMI) was performed using the formula of body weight divided by height^2^.

The patients participating in the trial provided written informed consent. The institutional ethics committee granted approval for the study procedure under the principles outlined in the Declaration of Helsinki. No artificial intelligence-based tools or technologies were used in the study.

Exclusion Criteria: Patients presenting with acute coronary syndrome, malignancies, atrial fibrillation, connective tissue disorders, hepatic failure, renal failure, a documented history of cardiac pacemaker implantation, prior bypass surgeries or surgical valve replacements, cardiomyopathy, congenital heart anomalies, or significant stenosis surpassing 50% in a vessel other than the CTO were excluded from the study, as these conditions may be categorized as primary lesions that could adversely affect the outcomes of the investigation. Patients receiving pharmacological interventions that affect ventricular repolarization, other than beta-adrenergic antagonists, were systematically excluded.

Laboratory Measurements: Complete blood count and serum biochemistry tests were obtained from the patients. It was determined that individuals exhibiting abnormal electrolyte levels, particularly concerning serum potassium, sodium, and calcium concentrations, would be excluded at the time of hospital admission. Hemogram and biochemistry measurements were analyzed from peripheral venous blood samples post 12 h of fasting, and standard tubes were used in the analysis. Lipid panel, fasting glucose, and creatine were measured by the chemiluminescence method. Samples were collected into EDTA anticoagulated tubes to measure complete blood count. Samples were run on a Beckman Coulter analyzer, an automated blood cell counter.

Electrocardiogram: A 12-lead ECG (150 Hz filter, 25 mm/s, 10 mm/mV; Schiller, Cardiovit AT-10, Baar, Switzerland) was performed on the patients. All electrocardiograms analyzed in this investigation were conducted during outpatient clinic when the patients exhibited stable clinical conditions. The frontal QRS-T angle was calculated by recording the absolute value of the difference between the QRS angle and the T angle. The final value was calculated by subtracting the frontal QRS-T angle from 360° if it is 180° or above [7]. If the frontal QRS-T angle was determined to be ≥110° in men and ≥90° in women, the frontal QRS-T angle was considered to be increased, whereas the frontal QRS-T angle was accepted in the normal range if it measured <110° in men and <90° in women [15]. According to the mentioned evaluation, the patients were divided into two distinct categories as “increased frontal QRS-T angle” and “normal frontal QRS-T angle.” In the present investigation, the QRS–T angle thresholds of ≥110° for males and ≥90° for females delineate the normative distributions of the frontal QRS–T angle within the general population. Empirical research has indicated that the definitive reference value for the frontal QRS–T angle in individuals with chronic total occlusion remains inadequately defined. Consequently, reference values derived from studies conducted on the general population were employed for the purpose of hypothesis formulation and should not be construed as criteria for clinical decision making.

Transthoracic Echocardiography: Transthoracic echocardiography was performed by an experienced cardiologist using Philips Epiq 7 systems with 1 to 5 MHz X5-1 transducers (Andover, MA, USA). Echocardiography images were obtained from parasternal long axis, parasternal short axis, apical four-chamber, and apical two-chamber axis views with simultaneous ECG signal in the left decubitus position. Standard two-dimensional and color Doppler flow images were obtained under the guidelines of the American Society of Echocardiography [16]. Left ventricular ejection fraction was calculated by the modified Simpson’s method.

Coronary Angiography: Coronary angiography was performed by the Judkins method. The left anterior descending (LAD) and circumflex (CX) arteries were imaged from at least four different angles, and the right coronary artery (RCA) from at least two different angles. The lesion of patients with 100% occlusion on CAG and whose symptoms persisted for longer than three months was considered CTO [17]. In the study, vessels with chronic total occlusion were classified as LAD CTO, Cx CTO, and RCA CTO. Coronary collateral circulation was assessed by two experienced invasive cardiologists. In cases of disagreement, the images were re-examined by a third invasive cardiologist, and the final score was determined by reaching a consensus. Kappa coefficients were not calculated to measure interobserver reliability. The coronary CC of the vessel with CTO was evaluated using Rentrop Classification [18]. Rentrop 0 and Rentrop 1 were considered as poor collateral circulation, while Rentrop 2 and Rentrop 3 were considered as well-developed collateral circulation.

Statistical analysis: The analysis of the data was conducted using SPSS software (Version 23.0, SPSS, Inc., Chicago, IL, USA). It was evaluated whether the non-categorical data were normally distributed by the visual examination of the distribution (histograms, probability plots) and analytical method (Kolmogorov–Smirnov/Shapiro–Wilk’s test). Levine’s test was performed to evaluate homogeneity. The mean and standard deviation were used to report continuous variables, while percentages were used to report categorical variables in the table. Whether the categorical variables differed between the groups was evaluated using the chi-square or Fisher’s exact test. The means of normally distributed parameters were compared using the one-way ANOVA test. After comparing the groups with normal QRS-T axis and with abnormal QRS-T axis by univariate analysis, parameters that showed significant differences (*p* < 0.05) were evaluated with univariate logistic regression analysis to determine the factors predicting the group with abnormal QRS-T axis. The frontal QRS–T angle was used as the dependent variable and was classified such that an elevated QRS–T angle was assigned a value of 1, whereas a normative QRS–T angle was designated a value of 0. Owing to the constrained sample dimension (n = 120), the backward logistic regression technique was favored to circumvent overparameterization within the model and to more precisely evaluate the autonomous effects of clinically pertinent variables.

Multivariate logistic regression (backward method) analysis was performed for the parameters that were found to be significant in univariate logistic regression analysis, which were determined by the ‘*’ sign in Table 1. Finally, the sensitivity and specificity of poor collateral flow in detecting abnormal QRS-T angles were determined by ROC analysis.

## 3. Results

Patients (mean age of 58.1 years) were categorized into two groups based on their QRS-T angle, specifically those exhibiting increased values (>110° for men and >90° for women) and those demonstrating a normal QRS-T angle (Table 2). Female gender (*p* = 0.007), poor collateral flow (*p* < 0.001), furosemide usage (*p* = 0.020), oral anticoagulant usage (*p* = 0.020), mean age (*p* < 0.001), fasting glucose (*p* = 0.039), serum creatinine (*p* = 0.045), triglyceride (*p* = 0.003), white blood cell (*p* = 0.003), neutrophil (*p* < 0.001), left ventricular end-diastolic diameter (*p* < 0.001), and end-systolic diameter (*p* < 0.001) were higher in the group with increased QRS-T angle. Even if it did not reach statistical significance, hypertension (67.9% vs. 81%), diabetes (30.8% vs. 47.6%), and oral antidiabetic usage (24.4% vs. 40.5%) were higher in the group with increased QRS-T angle. Despite that, LVEF (*p* < 0.001), serum albumin (*p* = 0.001), and hemoglobin level (*p* = 0.002) were lower in this group. Furthermore, no statistically significant difference was observed among the groups with respect to the QRS–T angle and the anatomical distribution of chronic total occlusion. The locations of chronic total occlusion are delineated in the demographic table (Table 2).

**Figure 1:**
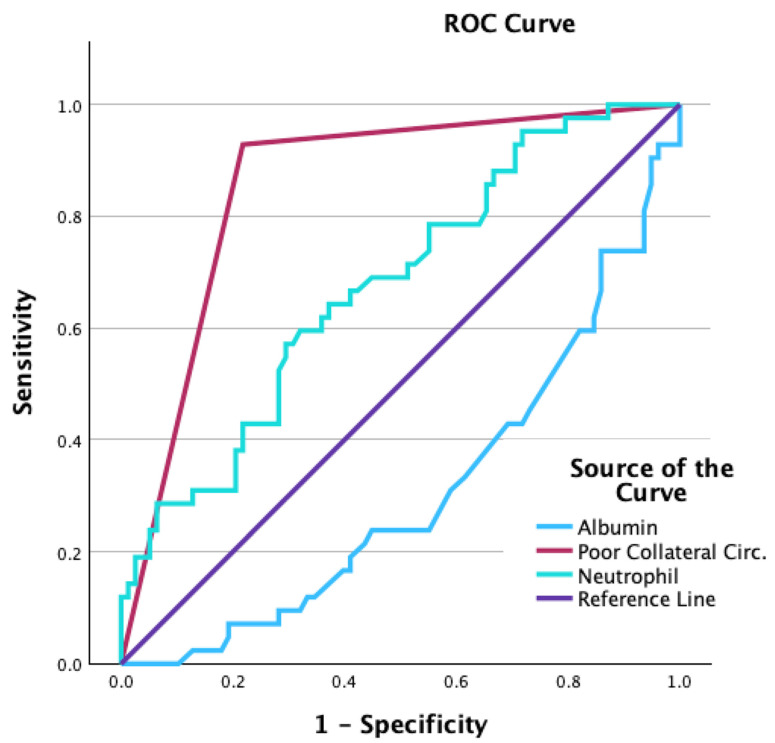
The sensitivity and the specificity of the presence of poor collaterals in predicting increased frontal QRS-T angle were 92% and 79%, respectively.

The parameters that showed statistically significant differences between the two groups in univariate analysis were evaluated with univariate logistic regression analysis (Table 1). In the course of conducting regression analysis, the parameters including furosemide, oral anticoagulants, fasting glucose, triglycerides, white blood cell count, left ventricular diastolic diameter, left ventricular systolic diameter, and left ventricular ejection fraction were systematically excluded. As a result, serum albumin level (OR = 0.711, 95% CI 0.564–0.896; *p* = 0.004), serum neutrophil level (OR = 1.478, 95% CI 1.080–2.022; *p* = 0.10), and poor collateral flow (OR = 17.7, 95% CI 12.2–85.3; *p* < 0.001) were found to be independent predictors of increased QRS-T angle (Table 3). The sensitivity and specificity of collateral detecting increased QRS-T angle by ROC analysis were AUC = 0.855, *p* < 0.001 and AUC = 0.693, *p* = 0.001, respectively (Figure 1).

## 4. Discussion

The present study showed that serum albumin level and poor collateral flow were independent predictors of increased frontal QRS-T angle in patients with CTO. We found that the presence of poor collateral flow increased the frontal QRS-T angle independently of low EF. This study represents the inaugural investigation in the existing body of literature that elucidates the correlation between the frontal QRS-T angle and the presence of poor collateral in cases of chronic total occlusion.

The frontal QRS-T angle is a marker of heterogeneity of myocardial repolarization and electrical instability of the myocardium. Recent research has indicated that the frontal QRS-T angle exhibits a robust and autonomous prognostication of mortality in individuals diagnosed with cardiovascular disease as well as among the general population of healthy individuals [19]. Increased frontal QRS-T angle was found to be an independent predictor of low FFR values, indicating ischemia in patients who underwent FFR evaluation for coronary artery stenosis [20]. The extent and severity of coronary artery disease in patients undergoing coronary angiography for stable angina are also linked to this phenomenon [21]. Long-term survival improves after CTO revascularization. The decrease in arrhythmic burden is one of the most crucial reasons for this improvement. Uzun et al. showed that successful percutaneous coronary intervention (PCI) can improve frontal QRS-T angle in patients with CTO [22]. Ischemia causes partial depolarization and repolarization of the ventricle along with conduction delay in Purkinje fibers [23]. This is the primary reason for the deterioration of ventricular homogeneity of the myocardium. The widening of the frontal QRS-T angle in the presence of poor collateral seems to be due to the damaged areas of the myocardium. Studies showing the association of poor collateral presence with ventricular repolarization and electrical abnormalities stand out in the literature [24]. Nonetheless, the QRS-T angle has been documented to exhibit superior reliability and reproducibility, while demonstrating reduced sensitivity to definitional challenges in comparison to alternative conventional electrocardiographic parameters that indicate ventricular repolarization and electrical irregularities [25,26]. The objective of the investigation was to assess the correlation between the frontal QRS–T angle and collateral circulation in individuals suffering from chronic total occlusion. As well, other established indicators of ventricular repolarization, specifically QTc, Tp–Te, and the Tp–Te/QT ratio, were not included in the analysis.

Sweda et al. revealed that the frequency of chronic renal failure was higher in patients with a widened frontal QRS-T angle [27]. In a study investigating the effect of age on ECG parameters, Giovanardi et al. found a correlation of increased frontal QRS-T angle with advanced age [28]. Sakhnova et al. found a significant relationship between low left ventricular ejection fraction and increased frontal QRS-T angle, highlighting the potential implications of cardiac function on electrocardiographic metrics [29]. In the current study, the correlation of the frontal QRS-T angle and the CC was in line with these findings.

Serum albumin functions as a detrimental acute-phase reactant in the process of inflammation [30]. Low serum albumin level is associated with cardiovascular disease and is an independent marker of adverse cardiac events [31]. Chen et al. found a positive correlation between albumin and well-developed coronary CC in CTO patients [32]. In another study in which elective PCI was performed, a relationship was demonstrated between serum albumin level and corrected QT interval on ECG [33]. Our investigation found that a low level of serum albumin was a separate factor that could predict a broad frontal QRS-T angle in patients with CTO. In a study, a wide QRS-T angle was found to be a marker of increased inflammatory activity independent of hypertension and diabetes [34].

Limitations: The study was conducted from a single center, with a relatively small population. This investigation examines the cross-sectional correlation between the frontal QRS–T angle and the extent of collateral circulation in individuals diagnosed with CTO. Our research did not explore temporal directionality or causative relationships. Consequently, the results merely represent concurrent associations and do not facilitate prognostic interpretations. Clinical outcomes, including arrhythmia, mortality, sudden cardiac death, and the progression of ventricular function, were not systematically gathered within the confines of the study. Consequently, the prognostic significance or treatment-determining capacity of the frontal QRS–T angle cannot be asserted based on the data currently at hand. In addition, measurement of electrolyte levels such as magnesium, which alter ventricular repolarization, and thyroid function tests would be more reasonable. One of the constraints inherent in our investigation is the omission of the kappa coefficient calculation in the assessment of interobserver reliability pertaining to the evaluation of collateral circulation in vessels exhibiting CTO.

## 5. Conclusions

The frontal QRS-T angle is a simple, easily accessible, and cost-effective parameter. It is more reliable, consistent, and less sensitive to miscalculation and misidentification than other conventional electrocardiographic myocardial repolarization parameters. Our findings suggest that an augmented QRS–T angle signifies repolarization variability attributable to collateral inadequacy; consequently, it may be regarded as a metric that can aid in and reinforce the Rentrop Classification in the assessment of angiographic poor collateral.

Therefore, revealing the bad collateral relationship with this ECG parameter may enable physicians to prevent the risk factors related to the widened QRS-T angle beforehand.

## Figures and Tables

**Table 1:** Univariate Binary Logistic Regression Analysis.

	OR	%95 CI Lower	%95 CI Upper	*p*
Age *	1.072	1.028	1.119	0.001
Gender *	0.235	0.080	0.692	0.009
Poor collaterals *	4.667	1.283	17.12	<0.001
Furosemide *	10.45	1.173	92.283	0.035
Oral Anticoagulant *	10.45	1.173	92.28	0.035
Glucose *	1.008	1.001	1.015	0.045
Serum creatinine	1.985	0.888	4.439	0.095
Serum Albumin *	0.102	0.025	0.424	0.002
Triglyceride *	0.990	0.984	0.997	0.006
White blood cell	1.276	1.080	1.508	0.004
Neutrophil *	1.381	1.144	1.669	<0.001
Hemoglobin *	0.742	0.608	0.905	0.003
LV Ejection Fraction *	0.914	0.875	0.954	<0.001
LV Diastolic diameter	1.185	1.091	1.287	<0.001
LV Systolic diameter	1.172	1.072	1.280	<0.001

LV: left ventricle. * Data identified as significant subsequent to the execution of univariate logistic regression analysis were subsequently subjected to multivariate logistic regression analysis.

**Table 2:** Demographic and clinical data.

	Normal QRS-T (n = 78)	Increased QRS-T (n = 42)	*p*
	**Demographic Data**		
Gender (w) n (%)	6 (7.7)	11 (26.1)	**0.007**
Age (years)	61.7 ± 8.7	68.2 ± 11.1	**<0.001**
Hypertension n (%)	53 (67.9)	34 (81)	0.094
Hyperlipidemia n (%)	35 (44.9)	17 (40.5)	0.394
Diabetes Mellitus n (%)	24 (30.8)	20 (47.6)	0.052
BMI (kg/m^2^)	29.9 ± 4.5	28.6 ± 4.4	0.120
Smoking n (%)	33 (42.3)	23 (54.8)	0.133
History of PCI n (%)	43 (55.1)	22 (52.4)	0.461
Poor collaterals n (%)	18 (23.1)	40 (95)	**<0.001**
Frontal QRS-T Angle (°)	43.8 ± 31.2	139.4 ± 23.8	**<0.001**
	**Drug Usage**		
Acetyl salicylic acid n (%)	52 (66.7)	24 (57.1)	0.202
P2Y12 Inhibitors n (%)	5 (6.4)	2 (4.8)	0.531
ACE Inhibitors n (%)	27 (34.6)	16 (38.1)	0.424
ARB n (%)	18 (23.1)	12 (28.6)	0.326
Beta Blocker n (%)	32 (41)	20 (47.6)	0.307
Calcium Channel Blocker n (%)	12 (15.4)	7 (16.7)	0.524
Furosemide n (%)	1 (1.3)	5 (11.9)	**0.020**
Statin n (%)	19 (24.4)	12 (28.6)	0.385
Oral Antidiabetic n (%)	19 (24.4)	17 (40.5)	0.053
Insulin n (%)	6 (7.7)	4 (9.5)	0.488
Oral Anticoagulant n (%)	1 (1.3)	5 (11.9)	0.020
LAD CTO n (%)	13 (16.7)	8 (19)	0.463
Circumflex CTO n (%)	17 (21.8)	9 (21.42)	0.579
RCA CTO n (%)	48 (61.5)	26 (61.9)	0.564
	**Laboratory Data**		
Glucose (mg/dL)	121.2 ± 43.5	141.3 ± 61.2	0.039
Serum Creatinine (mg/dL)	1.01 ± 0.37	1.25 ± 0.93	0.045
Albumin (g/dL)	4.18 ± 0.28	3.95 ± 0.64	**0.001**
Total Cholesterol (mg/dL)	206.1 ± 55.3	194.2 ± 38.4	0.202
Low-density lipoprotein (mg/dL)	136.6 ± 45.4	125.1 ± 36.3	0.158
High-density lipoprotein (mg/dL)	43.1 ± 7.8	44.1 ± 11.1	0.347
Triglyceride (mg/dL)	174 ± 77	133 ± 59.1	**0.003**
WBC (10^9^/L)	8.44 ± 2.33	9.82 ± 2.33	**0.003**
Neutrophil (10^9^/L)	5.4 ± 1.9	6.9 ± 2.47	**<0.001**
Lymphocyte (10^9^/L)	2.2 ± 0.82	2.02 ± 1.1	0.236
Hemoglobin (g/dL)	14.4 ± 1.8	13.3 ± 2.15	**0.002**
	**Echocardiographic Data**		
LV Ejection Frac (%)	52.1 ± 8.2	43.2 ± 11.2	**<0.001**
LV Diastolic diameter (mm)	49.2 ± 3.5	53.4 ± 7.2	**<0.001**
LV Systolic diameter (mm)	33.5 ± 3.6	39.8 ± 8.9	**<0.001**
Septum (mm)	11.8 ± 1.4	12.1 ± 2.1	0.593
Posterior Wall (mm)	10.8 ± 0.9	11.1 ± 1.4	0.460

ACE: angiotensin converting enzyme, ARB: angiotensin receptor blocker, BMI: body mass index, CTO: coronary total occlusion, LAD: left anterior descending, LV: left ventricle, PCI: percutaneous coronary intervention, RCA: right coronary artery, and WBC: white blood cell.

**Table 3:** Area Under the ROC Curve.

Variable(s)	Area	Std. Error	Asymptotic Sig.	Asymptotic 95% Confidence Interval	
				Lower Bound	Upper Bound
Poor Collateral Circ.	0.855	0.037	0.000	0.784	0.927
Albumin	0.307	0.050	0.000	0.210	0.404
Neutrophil	0.675	0.050	0.001	0.576	0.774

Power analysis results: With the current sample (n_0_ = 64, n_1_ = 56), effect size: d = 1.84, group ratio: n_1_/n_0_ ≈ 0.875, α = 0.05, two-sided calculated statistical power: Power ≈ 1.00 (≈100%).

## Data Availability

The datasets generated and/or analyzed during the current study are not publicly available due to ethical and privacy restrictions but are available from the corresponding author upon reasonable request.
